# A novel *NPM1-RARG-NPM1* chimeric fusion in acute myeloid leukaemia resembling acute promyelocytic leukaemia but resistant to all-trans retinoic acid and arsenic trioxide

**DOI:** 10.1038/s41416-019-0456-z

**Published:** 2019-04-18

**Authors:** Xue Chen, Fang Wang, Yang Zhang, Wen Teng, Panxiang Cao, Xiaoli Ma, Mingyue Liu, Yaoyao Tian, Tong Wang, Daijing Nie, Jing Zhang, Hongxing Liu, Wei Wang

**Affiliations:** 1 Division of Pathology & Laboratory Medicine, Hebei Yanda Lu Daopei Hospital, 065201 Langfang, China; 2 Department of Hematology, The 2nd Affiliated Hospital of Harbin Medical University, 150001 Harbin, China; 3 Beijing Lu Daopei Institute of Hematology, 100076 Beijing, China; 4 Division of Pathology & Laboratory Medicine, Beijing Lu Daopei Hospital, Beijing, 100076 China

**Keywords:** Acute myeloid leukaemia, Molecular medicine, Acute myeloid leukaemia

## Abstract

The *RARG* gene is a member of the nuclear hormone receptor superfamily and shares high homology with *RARA* and *RARB*. *RARA* is involved in translocation with *PML* in acute promyelocytic leukaemia (APL). Little is known about *RARB* or *RARG* rearrangement. *RARG* fusions were reported in only five APL patients and the partner genes were *NUP98*, *PML* and *CPSF6*. Here, we report *NPM1* as a new partner gene of *RARG* and identify a unique *NPM1-RARG-NPM1* chimeric fusion for the first time in an old male with morphological and immunophenotypical features of hypergranular APL but lacking response to all-trans retinoic acid (ATRA) and arsenic trioxide (As_2_O_3_) therapy. The structural features of the fusion transcript may account for the clinical resistance of the patient. *RARG* fusion is rare but recurrent in APL, further investigation in larger cohorts is expected to assess frequency, clinical characteristics and outcomes of *RARG*-translocation in APL.

## Background

Acute promyelocytic leukaemia (APL) is characterised by the *PML-RARA* fusion caused by t(15;17)(q22;q12) translocation. Rarely, APL cases carry gene fusions involving *RARG*, which is a member of the same retinoid acid receptor (*RAR*) family and shares high homology (90%) with *RARA* and *RARB*. In 2011, Such et al. reported the first APL case harbouring a rearrangement of *RARG*.^1^ To date, *NUP98-RARG*, *PML-RARG* and *CPSF6-RARG* translocations have been reported in a total of five acute myeloid leukaemia (AML) patients resembling APL.^1–4^ Here, we describe the first case with a novel *NPM1-RARG-NPM1* chimeric fusion in an old male with morphological and immunophenotypical features of hypergranular APL but lacking response to all-trans retinoic acid (ATRA) and arsenic trioxide (As_2_O_3_) therapy.

## Methods

### Case reports

A 69-year-old man was admitted because of 2-week history of asthenia and dizziness. Blood tests showed haemoglobin level of 123 g/L, platelet count of 204 × 10^9^/L, and white blood cell count of 1.5 × 10^9^/L. Fibrinogen, fibrin degradation products and D-dimer levels were 1.72 g/L (reference, 2.00–4.00 g/L), 20 μg/ml (reference, 0–5.0 μg/ml) and 5.25 μg/ml (reference, 0–0.23 μg/ml). Prothrombin time and activated partial thromboplastin time were 12.4 s (reference, 8.9–13.3 s) and 32.2 s (reference, 25.0–45.0 s), respectively.

Morphologic examination of bone marrow (BM) smears disclosed infiltration by 56% of hypergranular promyelocytes (Fig. S1a). These cells demonstrated strong and diffuse reactivity to myeloperoxidase cytochemical staining, which often covered the nucleus and consistent with the characteristics of APL (Fig. S1b). The blast cells were positive for CD13, CD33, CD45, CD9, CD64 and cytoplasmic myeloperoxidase, partially positive for HLA-DR, CD117, CD56 and CD123, but negative for CD34, CD14, CD 38, CD11b, CD16 and other T- or B-lymphoid related markers.

The chromosome karyotype was normal and t(15;17)(q22;q12) translocation was not detected by karyotyping (Fig. S1c). Multiplex-nested reverse transcription polymerase chain reaction (RT-PCR) designed to amplify 36 fusion transcripts, including *PML-RARA*, *ZBTB16-RARA* and *NPM1-RARA* showed 3 abnormal positive bands in one reaction which was designed to amplify *NPM1-RARA*. Sanger sequencing of PCR products revealed *NPM1-RARG* fusions of *NPM1* exon 4 to *RARG* partial exon 1, exon 2 or exon 4, respectively (Fig. S1d). The extensive homology between *RARA* and *RARG* made it possible to amplify *NPM1-RARG* using primers designed to amplify *NPM1-RARA*.

The patient was treated with As_2_O_3_ (10 mg/d, days 1–34) and showed no response. Then he was switched to ATRA therapy (50 mg/d, days 35–70) after the confirmation of *NPM1-RARG* rearrangement. The *NPM1-RARG* transcripts remained positive and were highly expressed in both peripheral blood and BM samples of the patient during the course of treatment. He refused to receive chemotherapy and died 8 months after diagnosis.

### Whole genome sequencing

To clarify the genomic breakpoints in *NPM1* and *RARG*, 30× whole genome sequencing (WGS) was performed on genomic DNA of BM sample using HiSeq X Ten (Illumina, Inc., San Diego, CA) after approval by the ethics committee at the 2nd Affiliated Hospital of Harbin Medical University. Raw reads in fastq were pre-processed and controlled for quality using fastp, followed by rapid genome analysis using speedseq with default parameters. Structural variants were called from speedseq with default options and the next annotation tool was AnnotSV.

### Targeted next-generation sequencing and mutation analysis

Mutational hotspots or whole coding regions of 86 genes that are known to be frequently mutated in haematologic malignancies were sequenced using a targeted, multiplexed amplicon-based high-throughput sequencing protocol as we previously reported.^5^

## Results

Laboratory, morphology and immunophenotypic analysis of the patient suggested diagnosis of hypergranular APL. *IDH1* R132H and *SRSF2* P95_R102del mutations were identified.

**Fig. 1:**
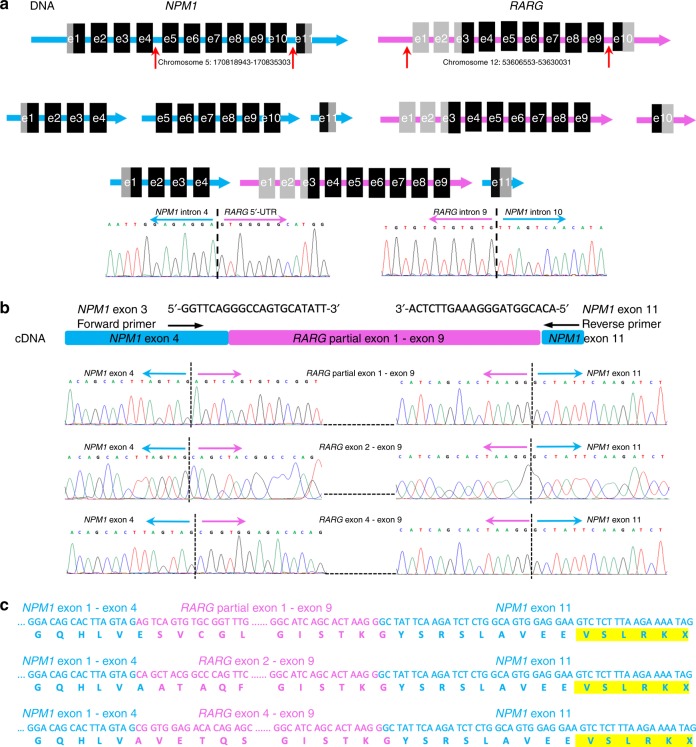
Identification of a novel *NPM1-RARG-NPM1* chimeric fusion in a APL case lacking t(15;17)(q22;q12)/*PML-RARA*. **a** WGS found that *NPM1* and *RARG* each has two breakpoints (shown in red arrows). Sequencing chromatogram showed the two genomic junction sequences (*NPM1* intron 4-*RARG* 5′-UTR and *RARG* intron 9- *NPM1* intron 10). **b** RT-PCR and sequencing of the PCR products verified the presence of *NPM1-RARG-NPM1* chimeric fusion with three kinds of fusion transcripts. **c** Expected protein sequences translated from the three fusion transcripts. The same oligomeric amino acid tail (VSLRK) as in C-terminal region of all mutant *NPM1* that frequently occurred in AML was shown in yellow

WGS analysis revealed breakpoints in intron 4 of *NPM1* and 5′ untranslated region (5′-UTR) of *RARG*. Interestingly, two more breakpoints in *NPM1* intron 10 and *RARG* intron 9 were identified. Both *NPM1* intron 4-*RARG* 5′-UTR and *RARG* intron 9-*NPM1* intron 10 genomic fusions were confirmed by Sanger sequencing. Hence the genomic alterations of this patient were a deletion of 16,360 bp of *NPM1* from intron 4 to intron 10 accompanied by an insertion of 23,479 bp of *RARG* from 5′ UTR to intron 9 (Fig. 1a). Moreover, RT-PCR and Sanger sequencing verified the presence of three *NPM1-RARG-NPM1* transcripts presumably derived from alternative splicing: *NPM1* (exon 1–4) — *RARG* (partial exon 1-exon 9) – *NPM1* (exon 11), *NPM1* (exon 1–4) – *RARG* (exon 2–9) – *NPM1* (exon 11) and *NPM1* (exon 1–4) – *RARG* (exon 4–9) – *NPM1* (exon 11) (Fig. 1b). The microdeletion of *NPM1* and microinsertion of *RARG* at the genomic level were too subtle to be found by karyotype analysis.

The *NPM1* 5′-region encoding the nucleoplasmin domain was fused to the DNA-binding domain (DBD) of *RARG* in all three transcripts. Deletion of *RARG* exon 10 led to 25 amino acids loss of the ligand-binding domain (LBD) of *RARG*. Notably, the three transcripts generated the same C-terminal oligomeric amino acid tail (VSLRK) as in all mutant *NPM1* that frequently occurred in AML^6^ due to the 3′ end fusion of *NPM1* exon 11 and frameshift coding. The two critical C-terminal tryptophan (W) residues at positions 288 and 290 which are necessary for nucleolar localisation of *NPM1* were also altered^6^ (Fig. 1c).

## Discussion

The *NPM1* gene encodes nucleophosmin, which is a highly conserved nucleo-cytoplasmic shuttling protein that shows restricted nucleolar localisation. Mutations or translocations involving *NPM1* gene cause cytoplasmic ectopia of nucleophosmin and are associated with several haematological malignancies, especially the bio pathogenesis of AML.^6^ *NPM1-RARA* has been reported as a very rare variant of *RARA* translocations in APL^7^ (Fig. S2a). In this case, the *NPM1-RARG-NPM1* fusion leads to both impairment of *NPM1* protein and abnormal of *RARG*. The missing of *NPM1* exon 5–9 and the mutation-like C-terminus of the *NPM1-RARG-NPM1* transcripts may result in impaired function and ectopia of nucleophosmin in cytoplasm and contribute to the impaired differentiation and leukogenesis.

*RARG*, *RARA* and *RARB* are nuclear hormone receptors functioning as ligand-dependent transcriptional activators that interact specifically to modulate transcription of DNA elements, and all have highly conserved DBD and LBD.^8^ Fusions and aberrations of *RARs* contributed to hematopoietic differentiation arrests at promyelocytes stage and constitute the basis for therapeutic response of ATRA-induced differentiation therapy. Although very rare, translocations involving *RARB* (*TBL1XR1-RARB*)^9^ and *RARG* (*NUP98-RARG*, *PML-RARG*, and *CPSF6-RARG*)^1–4^ have been reported in APL. As in *PML-RARA* and other *RARA* fusions, *RARB* and *RARG*-rearrangements in reported cases preserve both DBD and LBD^1–4,9^ (Fig. S2b, e). In the present case, deletion of *RARG* exon 10 caused 25 amino acids loss of LBD thus may result in impaired ATRA binding affinity (Fig. S2f). On the other hand, the fusion partner of *RARG* is *NPM1* rather than *PML* may make the patient resistant to As_2_O_3_ due to lack of As_2_O_3_ binding site.^10^ These are in line with the clinical resistance of ATRA and As_2_O_3_ of the patient.

## Conclusion

We report *NPM1* as a partner gene of *RARG* in a patient morphologically resembling APL but lacking response to ATRA and As_2_O_3_ therapy for the first time and identify a unique *NPM1-RARG-NPM1* chimeric fusion. *RARG* fusion with different partners is rare but recurrent in APL. Further investigation in larger cohorts is expected to assess frequency, clinical characteristics and outcomes of *RARG*-translocation in APL.

## Supplementary information

Identification of a novel NPM1-RARG-NPM1 chimeric fusion in a APL case lacking t(15;17)(q22;q12)/PML-RARA

## Data Availability

The datasets used and/or analysed during the current study are available from the corresponding author on reasonable request.

## References

[1] Such E, Cervera J, Valencia A, Barragan E, Ibanez M, Luna I (2011). A novel NUP98/RARG gene fusion in acute myeloid leukemia resembling acute promyelocytic leukemia. Blood.

[2] Ha JS, Do YR, Ki CS, Lee C, Kim DH, Lee W (2017). Identification of a novel PML-RARG fusion in acute promyelocytic leukemia. Leukemia.

[3] Liu T, Wen L, Yuan H, Wang Y, Yao L, Xu Y (2018). Identification of novel recurrent CPSF6-RARG fusions in acute myeloid leukemia resembling acute promyelocytic leukemia. Blood.

[4] Qin YZ, Huang XJ, Zhu HH (2018). Identification of a novel CPSF6-RARG fusion transcript in acute myeloid leukemia resembling acute promyelocytic leukemia. Leukemia.

[5] Zhang Y, Wang F, Chen X, Zhang Y, Wang M, Liu H (2018). CSF3R Mutations are frequently associated with abnormalities of RUNX1, CBFB, CEBPA, and NPM1 genes in acute myeloid leukemia. Cancer.

[6] Falini B, Mecucci C, Tiacci E, Alcalay M, Rosati R, Pasqualucci L (2005). Cytoplasmic nucleophosmin in acute myelogenous leukemia with a normal karyotype. N. Engl. J. Med..

[7] Kikuma T, Nakamachi Y, Noguchi Y, Okazaki Y, Shimomura D, Yakushijin K (2015). A new transcriptional variant and small azurophilic granules in an acute promyelocytic leukemia case with NPM1/RARA fusion gene. Int. J. Hematol..

[8] Marinelli A, Bossi D, Pelicci PG, Minucci S (2009). Redundant function of retinoic acid receptor isoforms in leukemogenesis unravels a prominent function of genome topology and architecture in the selection of mutagenic events in cancer. Leukemia.

[9] Osumi T, sujimoto SI, Tamura M, Uchiyama M, Nakabayashi K, Okamura K (2018). Recurrent RARB translocations in acute promyelocytic leukemia lacking RARA translocation. Cancer Res..

[10] Tomita A, Kiyoi H, Naoe T (2013). Mechanisms of action and resistance to all-trans retinoic acid (ATRA) and arsenic trioxide (As_2_O_3_) in acute promyelocytic leukemia. Int. J. Hematol..

