# Supplementary material for: A novel NPM1-RARG-NPM1 chimeric fusion in acute myeloid leukaemia resembling acute promyelocytic leukaemia but resistant to all-trans retinoic acid and arsenic trioxide
Source: Br J Cancer. 2019 Apr 18;120(11):1023–5. doi: 10.1038/s41416-019-0456-z (PMC6738072; doi:10.1038/s41416-019-0456-z)
Supplement: Supplementary file 1 — Identification of a novel NPM1-RARG-NPM1 chimeric fusion in a APL case lacking t(15;17)(q22;q12)/PML-RARA [file 41416_2019_456_MOESM1_ESM.docx]

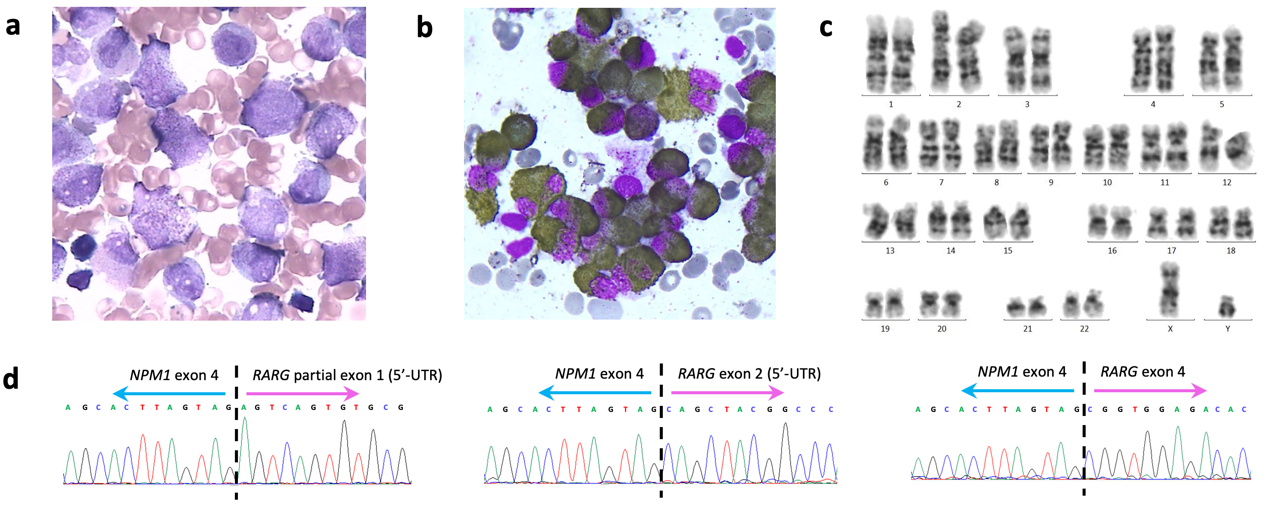


**Figure S1.** Morphology, karyotyping and multiplex-nested RT-PCR analysis. (**a**) Wright-Giemsa stained bone marrow smear showing several abnormal promyelocytes with hypergranulated cytoplasm. (**b**) The peroxidase stained bone marrow smear showed strong positivity. (**c**) G-banded karyotype showing 46, XY. (**d**) Multiplex-nested RT-PCR showed 3 abnormal positive bands in one reaction which was designed to amplify different isoforms of *NPM1-RARA*. Sequencing chromatogram of the PCR products revealed *NPM1-RARG* transcripts derived from fusion of *NPM1* exon4 to *RARG* partial exon 1, exon 2, or exon 4, respectively.

**a
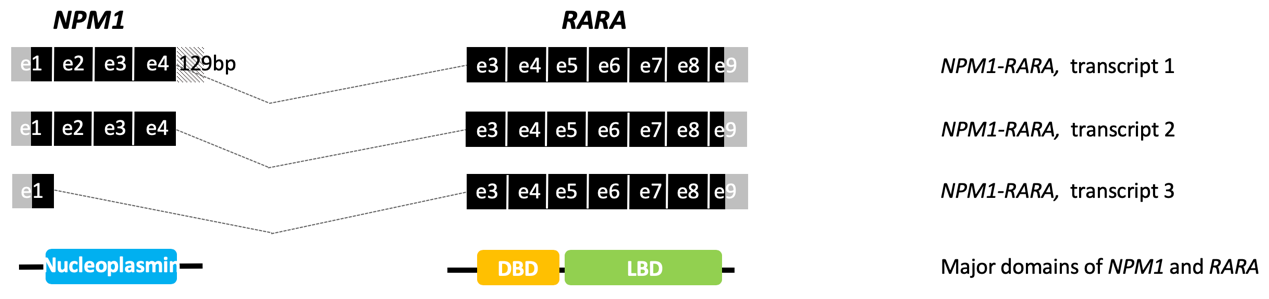
**

**b
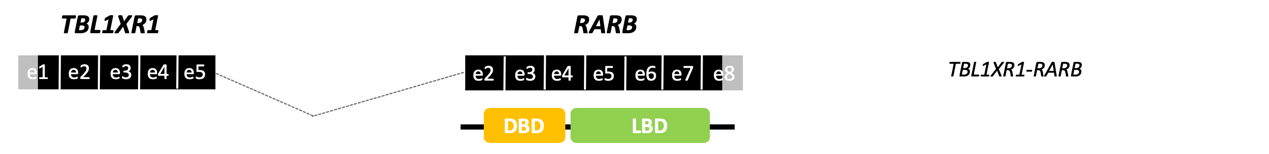
**

**c
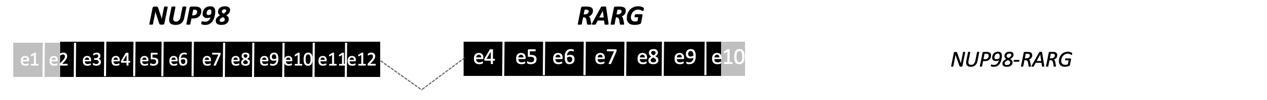
**

**d
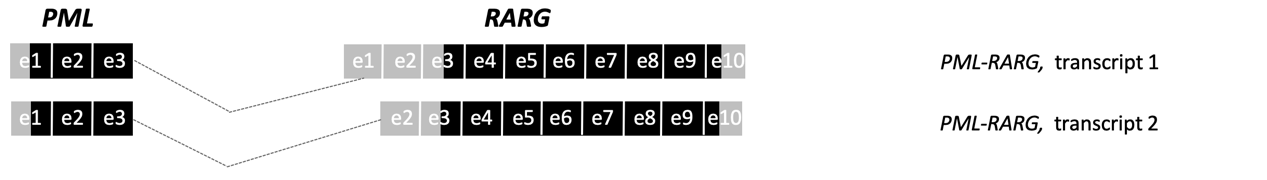
**

**e
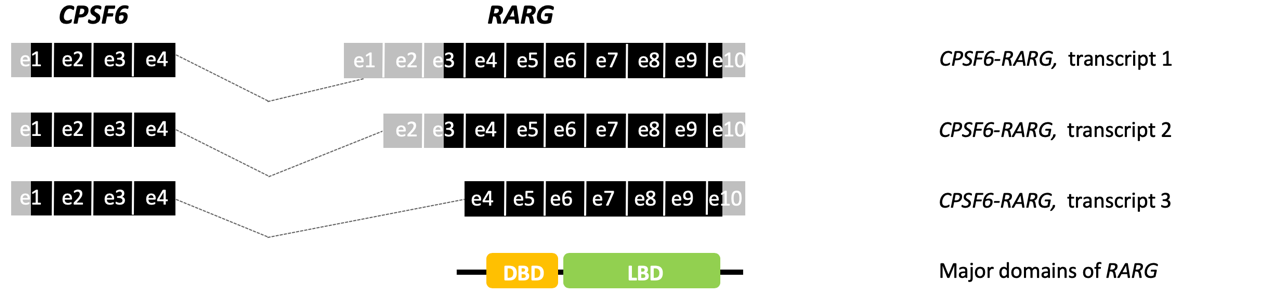
**

**f
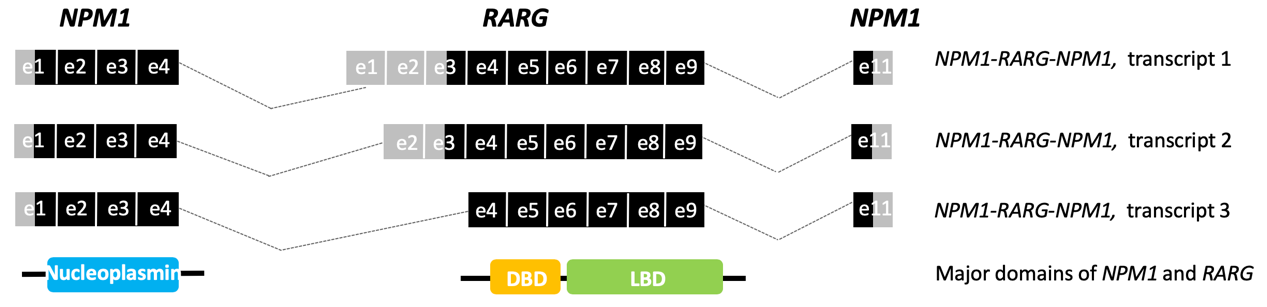
**

**Figure S2.** Schematic comparison of reported *NPM1*-translocations and *RARG*-rearrangements in APL and the *NPM1-RARG-NPM1* fusion in this case. (**a**) *NPM1* N-terminal nucleoplasmin domain is fused to the C-terminus of *RARA* in most *NPM1-RARA* fusions reported. (**b**) Schematic representation of the *TBL1XR1-RARB* fusion protein. (**c**) Schematic representation of the *NUP98-RARG* fusion protein. (**d**) Schematic representation of the *PML-RARG* fusion proteins. (**e**) Schematic representation of the *CPSF6-RARG* fusion proteins. (**f**) Schematic representation of the *NPM1-RARG-NPM1* fusion proteins in the present case. In all these transcripts, the *NPM1* 5’-region encoding the nucleoplasmin domain was fused to the DNA-binding domain (DBD) of RARG. Deletion of exon 10 of *RARG* led to the loss of 25 amino acids of the ligand-binding domain (LBD) of *RARG*.
